# Mechanism design of incentive-based reverse auctions with loss-averse 3PLs under incomplete attributes

**DOI:** 10.1371/journal.pone.0207937

**Published:** 2018-11-28

**Authors:** Xiaohu Qian, Min Huang, Qingyu Zhang, Mingqiang Yin, Xingwei Wang

**Affiliations:** 1 College of Management, Research Institute of Business Analytics & Supply Chain Management, Shenzhen University, Shenzhen, Guangdong, China; 2 College of Information Science and Engineering, Northeastern University, Shenyang, Liaoning, China; 3 State Key Laboratory of Synthetical Automation for Process Industries (Northeastern University), Shenyang, Liaoning, China; 4 College of Software, Northeastern University, Shenyang, Liaoning, China; Shandong University of Science and Technology, CHINA

## Abstract

As a supply chain solution integrator, fourth party logistics (4PL) has become an important focus for improving the operational efficiency of the logistics industry in recent days. This paper addresses the mechanism design problem of the 4PL for selecting a third party logistics (3PL) provider who involves loss-averse behavior to form a longer-term strategic partnership in multi-attribute reverse auctions. Due to fluctuating costs of energy or labor and unintentional delivery failures like traffic jam or technology malfunctions, we consider two incomplete attributes, namely cost uncertainty and delivery risk. Integrating the loss-averse behavior of 3PLs, based on the prospect theory, the bid decision model is constructed to obtain 3PLs’ bidding strategies. The corresponding efficient and optimal scoring auctions that consist of cost-sharing contract and contingent penalty are developed to maximize the ex ante expected profit of the system or the 4PL depending on whether the 4PL is willing to cooperate or not. Theoretical analysis verified by numerical examples illustrates the advantage of the proposed mechanisms. Impacts of model parameters on the 4PL’s decision are also investigated and managerial insights are presented.

## Introduction

With the development of economic globalization, logistics becomes a strategic focus of many companies for creating and delivering high value services. For example, companies like Xerox, Dell and Zara have achieved great success in the marketplace by investing significantly in developing logistics [1]. However, due to the increasing complexity of supply chain network, third party logistics (3PL) providers who provide a range of logistics activities may not satisfy expectations of their clients [2]. Hence, it becomes imperative for 3PLs to align with excellent consulting companies, advanced technology firms and other business process management enterprises to provide the cost-effective and sustainable supply chain solutions. This produces a lead logistics organization called fourth party logistics (4PL) providers.

The idea of 4PL was originated by the consulting company Accenture that defines a 4PL as a supply chain integrator that assembles the resources, capabilities, and technology of its own organization and other organizations to design, build and run comprehensive supply chain solutions [1]. It is reported that by applying the 4PL model, Accenture has transformed the logistics function of Unilever Southern Africa to increase the efficiency of the supply chain and to reduce carbon emissions [2]. In China, Adage Logistics, a solution integrator company, has positioned itself as a 4PL facilitator to provide services for manufacturers, retailers, and distributors to manage and execute their supply chain and logistics activities (http://www.adagelogistics.com). Also, Eternal Asia, a Chinese logistics and supply chain management firm, could connect brand enterprises, logistic service providers, financial institutions, value-added service providers and various organizations to offer integrated supply chain solutions for its clients (http://www.eascs.com/en/).

This study arises from the practical problems faced by a 4PL provider who seeks to adopt a multi-attribute reverse auction (MARA) for selecting a 3PL to form a longer-term strategic partnership. MARA is a traditional auction in a reverse format that allows the 4PL and 3PLs to negotiate over the price and non-price attributes [3, 4]. In such auctions, the 4PL provider announces the scoring rule to solicit bids from a number of pre-qualified 3PLs. Then, 3PLs simultaneously submit bids to the 4PL and the one with the lowest score wins the auction. On one hand, because of the uncertainties that surround the process of logistics and the potential changes in input costs such as energy or labor, the cost of each 3PL is uncertain at the time of bidding. In this circumstance, 3PLs could reduce the future cost by exerting their efforts to re-engineer or re-optimize the processes that could impact the performance [5]. In the meanwhile, because of the cost uncertainty, 3PLs would involve loss averse behavior [6]. Specifically, regarding the expected value as a reference point, 3PLs will interpret the actual values as gains or losses relative the reference point and are more sensitive to losses than to absolutely commensurate gains [7, 8]. On the other hand, bad weather or other unintentional events like traffic jam or technology malfunctions may result in late delivery [9, 10], and other delivery failures like loss or damage of goods in transit may also lead to customer dissatisfaction [11]. In this case, the capability of each 3PL to handle the delivery risk becomes important for the 4PL to achieve the goal of customer satisfaction. Overall, the main challenge of this research is to face the 4PL’s mechanism design problem with loss averse 3PLs considering two incomplete attributes, namely, cost uncertainty and delivery risk.

To be specific, the cost uncertainty can be represented by a certain part called average cost plus a random variable called unpredictable cost [12]. To focus on the cost-reduction effort, the delivery risk is assumed to be unintentional for reasons out of the 3PL’s control [11]. Noting that 3PLs often hold knowledge of their financial status, experience or expertise, the average cost and delivery risk are assumed to be 3PLs’ private information. For the sake of convenience, we refer to the 4PL as *she* and a 3PL as *he* in the following discussion. The objective of the 4PL provider is to maximize the system profit or her own profit depending on whether the 4PL is willing to cooperate or not, when she faces multiple loss-averse 3PLs.

To solve the problem, we developed an incentive-based first-score sealed-bid reverse auction which consists of two phases: the reverse auction phase and the contracting phase. In the first phase, a scoring rule that is a function of the bid price and contingent penalty is constructed to characterize the 4PL’s preference. The 3PL with the lowest score wins the auction and receives a part of his own bid price at the end of the reverse auction. In the second phase, the 4PL offers a cost-sharing contract to induce the winning 3PL to make the cost-reduction effort. The winning 3PL would pay the contingent penalty to the 4PL if the delivery risk occurs, and could be paid a part of the final cost at the end of the contracting phase. If the 4PL maximizes the system profit, then the incentive-based mechanism is called *efficient scoring auction with cost-sharing contract* (ESA-CC). If the 4PL maximizes her own profit, then the incentive-based mechanism is referred to as *optimal scoring auction with cost-sharing contract* (OSA-CC).

Our work contributes to the logistics and supply chain management literature by investigating a 4PL’s mechanism design problem with two incomplete attributes, namely, cost uncertainty and delivery risk, when facing multiple loss-averse 3PLs. Based on the prospect theory, by integrating the contingent penalty and cost-sharing contract with reverse auctions, the novel ESA-CC and OSA-CC are proposed to solve the problem. Comparing with other known mechanisms, theoretical analysis verified by numerical results shows the effectiveness and applicability of the proposed mechanisms. We believe that our work could improve the operational efficiency of the 4PLs and benefit the logistics industry.

## Literature review

In the literature, a variety of auction mechanisms have been developed to purchase standard goods and services such as printers, computers and bookkeeping software, showing the growing interest and importance of employing auctions for procurement. Roughly, most of these studies assumed that the decision makers are prefectly rational, and concerned the auction models for complete attributes such as the certain cost and known quality without considering the incentive payment or the contingent penalty [13–16]. An excellent review of the literature can be available in [17]. Whereas adopting auctions for the procurement of customized goods and services to satisfy the buyer’s unique needs poses challenges due to the incomplete attributes such as cost uncertainty, supply risks and delivery disruptions [18–20]. The development of the auction models for incomplete attributes is very recent field of research that has increasingly received much attention in operations management. This paper investigates a 4PL provider’s transportation service procurement auction with multiple loss-averse 3PLs, and would pay an emphasis on the incomplete attributes to improve the procurement efficiency for the 4PL. Next, two separate streams related to the captured research topic would be reviewed, i.e., incentive-based reverse auctions and 4PL operations management.

In the literature of the incentive-based reverse auction, two types of policies could be adopted to deal with a single incomplete attribute, i.e., the incentive policy and the penalty policy. The first one focuses on the development of auction mechanisms with incentive policies. To be specific, the class of problems can be addressed by developing an incentive-based payment function for buyers to induce the cost-reduction efforts of bidders in the framework of conventional auctions. An initial example is the investigation of an optimal auction mechanism that combines a linear contract with the competitive bidding format proposed by McAfee & McMillan [12] to deal with the cost uncertainty such that the expected procurement cost can be minimized. In the subsequent works, Laffont & Tirole [21] extended the incentive-based payment function to a project procurement auction with an incomplete attribute of uncertain quality. It showed that the auction mechanism integrating an incentive contract could be better than the traditional one to handle the incomplete attribute. To release the full potential of the reverse auction, Che [22] extended Laffont & Tirole’s work to the two-attribute scenario, and proposed a general scoring-rule based reverse auction mechanism under which the revenue equivalence theorem still holds. Variations on the basic incentive-based auction model have subsequently been proposed to foster competition [5] or to promote collaboration [23]. The second one focuses on the development of auction mechanisms with penalty policies. These studies integrated a contingent penalty into the payment function to propose a revised auction mechanism for the buyer to manage the incomplete attribute. For example, when suppliers’ reliability is the main focus, introducing the contingent payment to the first-score sealed-bid reverse auction, Chen et al. [24] firstly proposed the efficient and optimal scoring mechanisms for the public and private sectors, respectively, and showed the distinct advantage of the mechanism by comparing it with other known mechanisms. Subsequently, based on the revelation principle, Chaturvedi & Martínez-de-Albéniz [11] employed the contingent payment to develop an optimal reverse auction assuming that bidders would adopt the dominant-strategy equilibrium bidding strategy. In the subsequent works, variations on the basic auction model that integrates the contingent payment function have been developed for supplier qualification screening [25, 26] or promoting the buyer and seller honesty [27]. However, the above works only examine one incomplete attribute, and the proposed mechanisms cannot directly be applied to our research problem that considers two incomplete attributes. We are trying to demonstrate how incentive policies and penalty policies can be combined with auction mechanisms to derive the optimal or efficient procurement strategies for the 4PL.

In the literature of 4PL operations management, many articles focus on the research problems at the tactical level under the scenario of symmetric information. These studies constructed mathematical models and developed solution methods to help the 4PL make the best decision. For example, an analytical multi-attribute decision making framework was constructed to evaluate the 4PL operating models for a logistics company that is willing to expand its operations [28]. In recent days, to identify the cost-effective ways of increasing the operational efficiency of logistics, a variety of interesting issues have been investigated, such as the routing problem [9, 19, 29, 30] and the network design problem [2, 31–33]. The above models developed so far assumed that the 4PL could have full information during the decision-making process. Yet, in practical applications, the 4PL may also have to make decisions under the scenario of asymmetric information. In this case, a game theoretical framework that has generated an impressive volume of research contributions would be employed for the design of auction mechanisms [15, 34–37]. For example, Huang et al. [38] considered a one-dimensional mechanism design problem in which a 4PL faces only one 3PL and designed a menu contract to induce the 3PL to exert effort for improving the quality of delivery. These works assumed that bidders are perfectly rational and could make the optimal decision. However, a large quantity of empirical and theoretical studies have shown that people would be affected by personal experiences or emotions, and the actual decision results could often deviate from the theoretical results assuming prefect rationality, showing that bidders would involve boundedly rational behaviors in the decision-making process [7, 39, 40]. For example, Liu et al. [8] considered that 3PLs could involve the loss averse behavior when facing uncertain demand of logistics services, and established a bi-objective order allocation model to maximize the total subjective utility of 3PLs as well as to minimize the total cost of the 4PL. To the best of our knowledge, the existing models cannot be fully applied to our problem that involves two incomplete attributes and loss-averse 3PLs under the scenario of asymmetric information.

To address the research gap, we investigate a reverse auction in which one 4PL faces multiple loss-averse 3PLs with two incomplete attributes. Integrating the incentive policy and penalty policy into the scoring auction, we propose a novel ESA-CC and OSA-CC depending on whether the 4PL is willing to cooperate or not. Theoretical analysis verified by numerical experiments shows the distinct advantage of the proposed mechanism by comparing it with other known mechanisms.

## Problem description

We model the reverse auction problem of a 4PL who faces *n* (*n* ≥ 2) loss-averse 3PLs and seeks to select one to deliver the transportation services for customers. Noting that when the transportation service is assigned, it might not be delivered as required [9]. The delivery risk occurs because of bad weather, traffic jam, technology malfunctions, etc. In this paper, we assume that the delivery risk is out of the 3PL’s control [11]. For simplicity, we model that 3PLs could involve high or low delivery risk in the market, which is denoted by *q*_*h*_ and *q*_*l*_, 0 < *q*_*l*_ < *q*_*h*_ ≤ 1, respectively. In specific, each 3PL knows his own delivery risk (private information), and believes that the probability of delivery risk of other 3PLs associated with *q*_*l*_ is *α* and with *q*_*h*_ is 1 − *α*, 0 ≤ *α* ≤ 1. If the delivery risk occurs or the customer’s requirement fails to be satisfied, then 3PL *i* would pay a contingent penalty *t*_*i*_ to the 4PL. In addition, due to uncertainties of the transportation process and changes of the energy or labor cost, the delivery cost of each 3PL is uncertain at the time of bidding. The uncertain cost can be characterized by a certain term called expected cost plus an uncertain term called unpredictable cost. The expected cost of 3PL *i* is denoted by *c*_*i*_ which is only known to the 3PL (private information). The cumulative distribution functions of the high and low delivery risk 3PLs are *F*_*h*_(*c*) and *F*_*l*_(*c*) with probability density functions *f*_*h*_(*c*) and *f*_*l*_(*c*) on the support $\left[\underline{c},\bar{c}\right]$, respectively. Obviously, we could see that $F_{h}\left(c\right)=\int_{\underline{c}}^{c}f_{h}\left(x\right)dx$ and $F_{l}\left(c\right)=\int_{\underline{c}}^{c}f_{l}\left(x\right)dx$. The unpredictable cost is denoted by a random variable *ϵ* which follows a normal distribution *G*(*ϵ*) with mean *μ* = 0, standard deviation *σ* > 0 and probability density function *g*(*ϵ*). 3PL *i* could exert cost-reduction effort *e*_*i*_ to reduce the unpredictable cost by re-engineering or re-optimizing the transportation process before he commences the transportation tasks. The dis-utility of the 3PL is characterized by a quadratic function *h*(*e*) = *ae*^2^ (see [41]), where *a* > 0 measures the challenge of cost reduction. In other words, 3PLs would spend more money to achieve the same cost-reduction goal with a higher *a*. Hence, the future cost of 3PL *i* could be denoted by *γ*_*i*_ = *c*_*i*_ + *ϵ* + *q*_*i*_*t*_*i*_ − *e*_*i*_.

The 4PL obtains value *v* if the delivered transportation service achieves the requirements of customers. Otherwise, the value is discounted by *z* and the 4PL only derives *v* − *z*. Noting that in the MARA system, 3PLs are required to submit the price-penalty combination (*b*_*i*_, *t*_*i*_), and could be evaluated by a scoring rule *S*(*b*, *t*) = *b* − Λ(*t*), where Λ(*t*) is an increasing concave function (i.e., Λ′(*t*) > 0 and Λ″(*t*)<0). For example, $\Lambda \left(t\right)=w\sqrt{t}$ represents a square-root scoring rule, where *w* measures the 4PL’s preference for the contingent penalty. The 4PL would pay *p* = *βγ* + (1 − *β*)*b* to the winning 3PL [12], where *β* ∈ [0, 1] is the cost-sharing parameter. If *β* = 0, the payment scheme defines a fixed-price contract, where the payment is simply the 3PL’s bid price. If 0 < *β* < 1, the payment scheme defines an incentive contract, where the payment depends on both the bid price and the future cost of the 3PL. If *β* = 1, the payment scheme defines a cost-plus contract, where the payment is simply the 3PL’s future cost. The purpose of the 4PL is to maximize the ex ante expected profit of the system or her own ex ante expected profit by appropriately determining the cost-sharing parameter *β* and the scoring rule parameter *w*.

**Fig 1:**
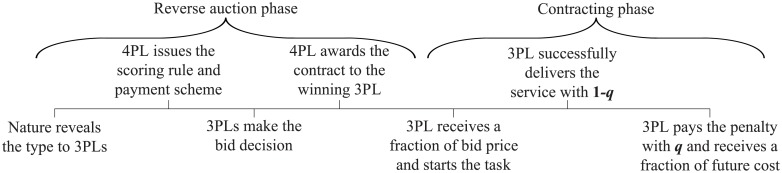
Timing of events.

In summary, the problem can be divided into two phases: the reverse auction phase and the contracting phase. In phase one, nature privately reveals the expected cost and delivery risk to each 3PL. Then the 4PL announces the scoring rule and the payment scheme before the bidding process. After that, 3PLs formulate their bids that include the bid price and contingent penalty to submit to the 4PL through the MARA system. The 3PL with the lowest score wins and is paid a fraction of the bid price, (1 − *β*)*b*, which concludes the reverse auction phase. In phase two, the winning 3PL firstly determines the optimal level of the cost-reduction effort to reduce the future cost. Then the 3PL would carry out the transportation tasks and could successfully make delivery with probability (1 − *q*_*i*_) because of delivery risk. If the transportation service fails to satisfy the requirement, then the 3PL pays the contingent penalty *t*_*i*_ to the 4PL with probability *q*_*i*_. Finally, the 4PL offers a fraction of the future cost *βγ* to the winning 3PL, and this concludes the contracting phase. The timing of events is shown in Fig 1.

Next we would solve the problem by working backward from the contracting phase. In other words, we would first derive the 3PLs’ optimal level of cost-reduction effort, and then analyze the bidding strategy of 3PLs, assuming that the 4PL has obtained the scoring rule and cost-sharing parameter in the reverse auction phase. We defer the discussion of the 4PL’s mechanism design problem to the later sections.

## Equilibrium analysis

This section presents the analysis of the decision of 3PLs’ equilibrium bidding strategies. Before detailed analysis, we introduce the definition of bidding strategy below.

Definition 1. *Given the scoring rule S*(*b*, *t*) *and the cost-sharing parameter β*, *the bidding strategy of a 3PL is defined as b*(⋅, ⋅, ⋅, ⋅; *β*), *which maps the expected cost* $c\in [\underline{c},\bar{c}]$, *delivery risk q* ∈ {*q*_*l*_, *q*_*h*_}, *contingent penalty t* ≥ 0 *and cost*-*reduction effort* $e\in [0,\frac{1}{2a}]$ *to a bid price*.

In this paper, 3PLs are assumed to be symmetric such that if they have the same ex ante expected cost and delivery risk, then they would exert the same level of cost-reduction effort and submit the same bid price. Next, we would consider a symmetric pure-strategy Bayesian-Nash equilibrium bidding strategy which maximizes the ex ante expected profit of each 3PL.

In the cost-sharing phase, if a 3PL wins the auction, then he needs to determine the optimal effort level to reduce the future cost. Given the payment scheme *p* = *βγ* + (1 − *β*)*b*, each 3PL chooses the effort level to maximize his own profit.

For any fixed loss-averse parameter λ > 1, based on the prospect theory, the ex post profit of a 3PL with loss-averse behavior can be expressed as:

$${\hat{\pi }}_{3PL}\left(e;b,t,c,q,\beta ,\epsilon \right)=\{\begin{array}{ll} \left(1-\beta \right)\left(b-c-qt+e-\epsilon \right)-h\left(e\right), & \text{if}\;\epsilon <0\\ \left(1-\beta \right)\left(b-c-qt+e-\lambda \epsilon \right)-h\left(e\right), & \text{if}\;\epsilon \geq 0 \end{array}$$ (1)

Taking the expectation of Eq (1) with respect to *ϵ*, the ex post expected profit of a 3PL could be expressed as:

$$\begin{array}{ccc} \pi_{3PL}\left(e;b,t,c,q,\beta \right) & = & \int_{-\infty }^{\infty }{\hat{\pi }}_{3PL}\left(e;b,t,c,q,\beta ,\epsilon \right)g\left(\epsilon \right)d\epsilon \\ & = & \left(1-\beta \right)(b-c-qt+e-\left(\lambda -1\right)\sigma /\sqrt{2\pi })-h\left(e\right). \end{array}$$ (2)

The optimal cost-reduction effort level of each 3PL denoted by e* can be obtained by maximizing Eq (2), which is shown in Lemma 1.

Lemma 1. *Given the cost-sharing parameter β*, *the optimal cost-reduction effort of a 3PL is denoted by* $e^{*}=\frac{1-\beta }{2a}$. *Moreover*, e* *is strictly decreasing in β*.

Note that all proofs are shown in Appendix 1. From Lemma 1, we observe that if a 3PL wins the auction, then his optimal cost-reduction effort e* is uniquely determined by the cost-sharing parameter *β*. Intuitively, when *β* increases, the ex post expected profit of 3PLs decreases. In this case, 3PL *i* would exert less effort to reduce the future cost. Hence, the optimal cost-reduction effort e* decreases as *β* increases.

In the reverse auction phase, each 3PL needs to firstly determine the contingent penalty if the delivery fails to meet the requirement. Let $\tau =\left(1-\beta \right)\left(b-c-qt-\left(\lambda -1\right)\sigma /\sqrt{2\pi }\right)+k^{*}\left(\beta \right)$ denote a 3PL’s expected profit, where $k^{*}\left(\beta \right)=\frac{{\left(1-\beta \right)}^{2}}{4a}$ is the extra amount of expected profit of the 3PL from his cost-reduction effort. Given any level of such supposed expected profit *τ* and delivery risk *q* ∈ {*q*_*l*_, *q*_*h*_}, to win the auction, the 3PL would choose the optimal contingent penalty t*(*q*) to minimize his score $S(b,\;t)=\left(\tau -k^{*}\left(\beta \right)\right)/\left(1-\beta \right)+c+qt+\left(\lambda -1\right)\sigma /\sqrt{2\pi }-\Lambda \left(t\right)$, i.e.,

$$t^{*}\left(q\right)=\underset{t}{\arg\min}\left\{S(b,\;t)\right\}.$$ (3)

Based on Eq (3), we can obtain the following Lemma 2.

Lemma 2. *Given the scoring rule S*(*b*, *t*), the optimal contingent penalty of each 3PL is t*(*q*) = Λ′^−1^(*q*). *Moreover*, t*(*q*) *is decreasing in q*.

From Lemma 2, we observe that the optimal contingent penalty t* is uniquely determined by the delivery risk. If a 3PL involves higher (lower) delivery risk, then he would place a smaller (larger) value of the optimal contingent penalty. In other words, given the scoring rule, all other things being equal, a 3PL with higher delivery risk would be scored higher and thereby would have fewer chances to win the auction. The quasi-linear scoring function can be adopted to effectively distinguish 3PLs in terms of their delivery risk.

Next, we would investigate the 3PLs’ equilibrium bidding strategies with respect to their ex ante expected costs. Given the form of the payment scheme and scoring rule, 3PLs with the same expected cost and delivery risk will choose the same bid price, *b*. Before detailed analysis, the definition of cost advantage is introduced below.

Definition 2. *Given the scoring rule S*(⋅, ⋅), *the cost advantage of a low delivery risk 3PL over a high one is defined as* $\Delta ≜S\left(\gamma ,t;q_{h}\right)-S\left(\gamma ,t;q_{l}\right)=\left[\Lambda \left(t^{*}\left(q_{l}\right)\right)-t^{*}\left(q_{l}\right)q_{l}\right]-\left[\Lambda \left(t^{*}\left(q_{h}\right)\right)-t^{*}\left(q_{h}\right)q_{h}\right]$.

From Definition 2, we know that Δ measures the cost advantage of a high delivery risk 3PL over a low one. Noting that the scoring rule parameter *w* can be uniquely determined according to the given Δ, we would focus on the determination of Δ in the following discussion. If $\Delta \geq \bar{c}-\underline{c}$, then the cost advantage of a low delivery risk 3PL is so distinct that the high delivery risk 3PLs can never win. To avoid trivial analysis, we assume that $\Delta <\bar{c}-\underline{c}$ in this paper.

Let $m\left(c\right)=\{\begin{array}{ll} c+\Delta , & c\leq c^{⋆}\\ \bar{c}, & c>c^{⋆} \end{array}$, where $c^{⋆}=\bar{c}-\Delta$. Based on Definition 2, we see that for any fixed $c\in [\underline{c},c^{⋆}]$, *S*(*b*(*c*, *q*_*l*_, t*(*q*_*l*_), e*), *q*_*l*_) = *S*(*b*(*m*(*c*), *q*_*h*_, t*(*q*_*h*_), e*), *q*_*h*_). To be specific, a low delivery risk 3PL with fixed cost $c\in [\underline{c},c^{⋆}]$ would be scored equal to the high delivery risk 3PL with fixed cost *m*(*c*). Based on the notations, the winning probabilities of 3PLs with *q*_*h*_ and *q*_*l*_ denoted by *θ*_*h*_(⋅) and *θ*_*l*_(⋅) could be expressed as:

$$\theta_{h}(c)={\left[\alpha (1-F_{l}(m(c)))+(1-\alpha )(1-F_{h}(c))\right]}^{n-1},$$ (4a)

$$\theta_{l}(c)={\left[\alpha (1-F_{l}(c))+(1-\alpha )(1-F_{h}(m^{-1}(c)))\right]}^{n-1}.$$ (4b)

From Eq (4a), we see that a high delivery risk 3PL wins the auction only if each of his competitors belongs to the high delivery risk group with expected cost higher than *c* or to the low delivery risk group with expected cost higher than *m*(*c*), which derives *θ*_*h*_(*c*). Similarly, from Eq (4b), we see that a low delivery risk 3PL wins the auction only if each of his competitors is a member of the high delivery risk group with expected cost higher than *m*^−1^(*c*) or a member of the low delivery risk group with expected cost higher than *c*, which derives *θ*_*l*_(*c*).

Based on Lemmas 1 and 2, given the expected cost $c\in [\underline{c},\bar{c}]$, delivery risk *q* ∈ {*q*_*l*_, *q*_*h*_}, contingent penalty *t* ≥ 0 and cost-reduction effort $e\in [0,\frac{1}{2a}]$ of a 3PL, the ex ante expected profit of the high or low delivery risk 3PLs could be derived as shown below:

$$\begin{array}{ccc} U_{H}\left(b;c,q_{h},t^{*}\left(\cdot \right),e^{*}\left(\cdot \right),\beta \right) & = & [\left(1-\beta \right)\left(b-c-t^{*}\left(q_{h}\right)q_{h}-\left(\lambda -1\right)\sigma /\sqrt{2\pi }\right)+k^{*}\left(\beta \right)]\theta_{h}\left(c\right), \end{array}$$ (5a)

$$\begin{array}{ccc} U_{L}\left(b;c,q_{l},t^{*}\left(\cdot \right),e^{*}\left(\cdot \right),\beta \right) & = & [\left(1-\beta \right)\left(b-c-t^{*}\left(q_{l}\right)q_{l}-\left(\lambda -1\right)\sigma /\sqrt{2\pi }\right)+k^{*}\left(\beta \right)]\theta_{l}\left(c\right). \end{array}$$ (5b)

Based on Eqs (4a), (4b), (5a) and (5b), the equilibrium bidding strategy that maximizes the ex ante expected profit of each 3PL is shown in Proposition 1.

Proposition 1. *Given the cost sharing contract p* = *βγ* + (1−*β*)*b and the scoring rule S*(*b*, *t*), *in equilibrium*, *3PLs choose the optimal cost-reduction effort as in Lemma 2*, *the optimal contingent penalty as in Lemma 2*, *and the Bayesian-Nash equilibrium bidding strategy as shown below*:

$$\begin{array}{ccc} b_{h}\left(c;q_{l},t^{*}\left(\cdot \right),e^{*}\left(\cdot \right),\beta \right) & = & c+q_{h}t^{*}\left(q_{h}\right)+\frac{\int_{c}^{\bar{c}}\theta_{h}\left(x\right)dx}{\theta_{h}\left(c\right)}-\frac{1-\beta }{4a}+\frac{(\lambda -1)\sigma }{\sqrt{2\pi }}, \end{array}$$ (6a)

$$\begin{array}{ccc} b_{l}\left(c;q_{l},t^{*}\left(\cdot \right),e^{*}\left(\cdot \right),\beta \right) & = & c+q_{l}t^{*}\left(q_{l}\right)+\frac{\int_{c}^{\bar{c}}\theta_{l}\left(x\right)dx+\int_{\bar{c}-\Delta }^{\bar{c}}\theta_{h}\left(x\right)dx}{\theta_{l}\left(c\right)}-\frac{1-\beta }{4a}+\frac{(\lambda -1)\sigma }{\sqrt{2\pi }}. \end{array}$$ (6b)

From Eqs (6a) and (6b), we find that the format of the bidding strategies for the high and low delivery risk 3PLs is similar, i.e., the expected cost plus the contingent penalty and information rent minus the cost improvement plus the additional cost induced by the uncertainty. For the high delivery risk 3PL, the information rent comes from the group of high delivery risk 3PLs. For the low delivery risk 3PL, the information rent comes from the group of both high and low delivery risk 3PLs. This is because the ex ante expected profit of a high delivery risk 3PL with $\bar{c}$ is zero, i.e., $U_{H}\left(b_{h};\bar{c},q_{h},t^{*}\left(\cdot \right),e^{*}\left(\cdot \right),\beta \right)=0$, but the ex ante expected profit of a low delivery risk 3PL with $\bar{c}$ is positive, i.e., $U_{L}\left(b_{l};\bar{c},q_{l},t^{*}\left(\cdot \right),e^{*}\left(\cdot \right),\beta \right)=U_{H}\left(b_{h};m^{-1}\left(\bar{c}\right),q_{h},t^{*}\left(\cdot \right),e^{*}\left(\cdot \right),\beta \right)>0$. In addition, when the loss-averse degree parameter λ = 1, the bidding strategy degenerates to the situation assuming loss-neutral 3PLs. Obviously, the 4PL pays more if 3PLs would involve loss-averse behaviors.

Next, the optimal ex ante expected profit of 3PLs is shown in the following corollary.

Corollary 1. *Given the cost sharing parameter β and the scoring rule S*(*b*, *t*), *for any fixed* $c\in [\underline{c},\bar{c}]$, *q*_*h*_, *q*_*l*_ ∈ (0, 1], *the optimal ex ante expected profit of a 3PL with c and q*_*h*_ *is* $U_{H}^{*}=\left(1-\beta \right)\int_{c}^{\bar{c}}\theta_{h}\left(x\right)dx$, *and the optimal ex ante expected profit of a 3PL with c and q*_*l*_ *is* $U_{L}^{*}=\left(1-\beta \right)\left[\int_{c}^{\bar{c}}\theta_{l}\left(x\right)dx+\int_{\bar{c}-\Delta }^{\bar{c}}\theta_{h}\left(x\right)dx\right]$.

Corollary 1 implies that 3PLs could not benefit from the loss-averse behavior since the extra cost, $\left(\lambda -1\right)\sigma /\sqrt{2\pi }$, is canceled out in calculation of the optimal ex ante expected profit. In addition, the optimal ex ante expected profit of 3PLs would decrease as the 4PL’s willingness to share the cost uncertainty increases. Next, the property of the bid price of 3PLs is examined.

Corollary 2. *Given* λ > 1, *b*_*h*_(*c*; *q*_*l*_, t*(⋅), e*(⋅), *β*) *and b*_*l*_(*c*; *q*_*l*_, t*(⋅), e*(⋅), *β*) *are increasing functions of* λ.

Corollary 2 shows that 3PLs would bid higher as the loss-averse degree is intensified. In this case, the 4PL would pay more to the winning 3PL. This indicates that the loss-averse behavior of 3PLs could have adverse effect on the 4PL’s expected profit.

## Efficient scoring auction with cost-sharing contract (ESA-CC)

In this section, we assume that the 4PL is willing to cooperate with 3PLs and acts as a Stackelberg leader to design ESA-CC for selecting a 3PL such that the ex ante expected profit of the system is maximized. In other words, the 4PL chooses a scoring function *S*(*b*, *t*) and the cost-sharing parameter *β* to maximize the total ex ante expected profit of herself and potential 3PLs by taking into account each 3PL’s best response.

Recall that the 4PL derives value *v* if the delivery succeeds, but otherwise obtains value *v* − *z* when the delivery fails to meet the requirements. Given the payment scheme *p* = *βγ* + (1 − *β*)*b*, the ex post expected profit of the 4PL is

$$\pi_{4PL}=E_{\epsilon }\left[v-p-qz+qt\right]=v-\left(1-\beta \right)\left(b-c-qt+e\right)-c-qz+e.$$

Obviously, the total ex post expected profit of the 4PL and the winning 3PL is

$$\pi_{T}=\pi_{3PL}+\pi_{4PL}=v-qz-c+e-h\left(e\right)-\left(1-\beta \right)\left(\lambda -1\right)\sigma /\sqrt{2\pi }.$$

Hence, based on Lemma 1, Lemma 2 and Proposition 1, the ex ante expected profit of the system can be expressed by

$$\begin{array}{ccc} \Gamma (\Delta ,\beta ) & = & n\alpha \int_{\underline{c}}^{\bar{c}}\left[v-c-q_{l}z+\left(1-\beta^{2}\right)/\left(4a\right)-\left(1-\beta \right)\left(\lambda -1\right)\sigma /\sqrt{2\pi }\right]\theta_{l}\left(c\right)f_{l}\left(c\right)dc\\ & & +n\left(1-\alpha \right)\int_{\underline{c}}^{\bar{c}}\left[v-c-q_{h}z+\left(1-\beta^{2}\right)/\left(4a\right)-\left(1-\beta \right)\left(\lambda -1\right)\sigma /\sqrt{2\pi }\right]\theta_{h}\left(c\right)f_{h}\left(c\right)dc\\ & = & v+\left(1-\beta^{2}\right)/\left(4a\right)-\left(1-\beta \right)\left(\lambda -1\right)\sigma /\sqrt{2\pi }\\ & & -n\alpha \int_{\underline{c}}^{\bar{c}}\left[c+q_{l}z\right]\theta_{l}\left(c\right)f_{l}\left(c\right)dc-n\left(1-\alpha \right)\int_{\underline{c}}^{\bar{c}}\left[c+q_{h}z\right]\theta_{h}\left(c\right)f_{h}\left(c\right)dc \end{array}$$ (7)

where the second equality is due to $n[\alpha \int_{\underline{c}}^{\bar{c}}\theta_{l}\left(c\right)f_{l}\left(c\right)dc+\left(1-\alpha \right)\int_{\underline{c}}^{\bar{c}}\theta_{h}\left(c\right)f_{h}\left(c\right)dc]=1$.

The 4PL chooses the optimal cost-sharing parameter *β*^*E*^ and cost advantage parameter Δ^*E*^ by maximizing Eq (7). The result is shown in the following proposition.

Proposition 2. *The ex ante expected profit of the system is maximized by setting* Δ^*E*^ = *z*(*q*_*h*_ − *q*_*l*_) *and* $\beta^{E}=2a\left(\lambda -1\right)\sigma /\sqrt{2\pi }$.

Proposition 2 implies that ESA-CC is independent of the distribution of 3PLs’ expected cost. Estimating the delivery risk *q*_*h*_, *q*_*l*_, standard deviation of unpredictable cost *σ* and parameters λ, *a*, the ESA-CC can be implemented and the ex ante expected profit of the system can be maximized.

Corollary 3. *In ESA*-*CC*, *β*^*E*^ *increases as the loss-averse parameter increases in* (1, ∞).

Corollary 3 implies that a 3PL would be less likely to be responsible for the cost uncertainty as the loss-averse degree becomes intensified. Recalling Corollary 1, we may infer that the loss-averse behavior of 3PLs could have adverse effect on bidders in terms of the ex ante expected profit.

## Optimal scoring auction with cost-sharing contract (OSA-CC)

When the 4PL are not willing to cooperate, she only tries to maximize her own ex ante expected profit, which is referred to as OSA-CC. This section presents the decision process of the 4PL for designing OSA-CC.

Based on the equilibrium bidding strategies obtained in the previous section, the 4PL’s ex ante expected profit could be obtained as shown below,

$$\begin{array}{ccc} ϒ(\Delta ,\beta ) & = & v+\left(1-\beta^{2}\right)/\left(4a\right)-\left(1-\beta \right)\left(\lambda -1\right)\sigma /\sqrt{2\pi }\\ & & -n\alpha \int_{\underline{c}}^{\bar{c}}[q_{l}z+c+\left(1-\beta \right)\frac{F_{l}\left(c\right)}{f_{l}\left(c\right)}]\theta_{l}\left(c\right)f_{l}\left(c\right)dc-n\alpha \left(1-\beta \right)\int_{\bar{c}-\Delta }^{\bar{c}}\theta_{h}\left(c\right)dc\\ & & -n\left(1-\alpha \right)\int_{\underline{c}}^{\bar{c}}[q_{h}z+c+\left(1-\beta \right)\frac{F_{h}\left(c\right)}{f_{h}\left(c\right)}]\theta_{h}\left(c\right)f_{h}\left(c\right)dc \end{array}$$ (8)

where the derivation of Eq (8) is given in Appendix 2. Maximizing Eq (8), we can obtain the following proposition.

Proposition 3. *The optimal cost advantage and cost*-*sharing parameters are characterized by the system of equations* (*first*-*order necessary condition*) *as shown below*

$$\int_{\underline{c}+\Delta }^{\bar{c}}[q_{h}z-q_{l}z-\Delta +\left(1-\beta \right)\frac{F_{h}\left(c-\Delta \right)}{f_{h}\left(c-\Delta \right)}-\left(1-\beta \right)\frac{F_{l}\left(c\right)}{f_{l}\left(c\right)}]\frac{d\theta_{l}\left(c\right)}{d\Delta }f_{l}\left(c\right)dc=\left(1-\beta \right)\theta_{h}\left(\bar{c}-\Delta \right),$$ (9a)

$$\alpha \int_{\bar{c}-\Delta }^{\bar{c}}\theta_{h}\left(c\right)dc+\alpha \int_{\underline{c}}^{\bar{c}}F_{l}\left(c\right)\theta_{l}\left(c\right)dc+\left(1-\alpha \right)\int_{\underline{c}}^{\bar{c}}F_{h}\left(c\right)\theta_{h}\left(c\right)dc+2a\left(\lambda -1\right)\sigma /\sqrt{2\pi }=\frac{\beta }{2an}$$ (9b)

*or at the corner point* Δ = 0 *and β* = 1.

Solving the system of equations defined by Eqs (9a) and (9b), and checking the corner points (*β*, Δ), the 4PL could derive the optimal cost advantage and cost-sharing parameters to maximize Eq (8). In the following discussion, the optimal cost advantage and cost-sharing parameters are denoted by Δ^*O*^ and *β*^*O*^, respectively. Next, by comparing the optimal parameters of ESA-CC and OSA-CC, we would provide additional suggestions for the 4PL to design such auctions. As a common assumption in auction literature [24], the distributional upgrade condition is introduced before detailed analysis.

Definition 3. *Given two cumulative distribution functions F*(*c*) *and* $\hat{F}\left(c\right)$ *with probability density functions f*(*c*) *and* $\hat{f}\left(c\right)$, for $c\in [\underline{c},\bar{c}]$, *F*(*c*) *is a distributional upgrade of* $\hat{F}\left(c\right)$ if $\frac{\hat{f}\left(c\right)}{\hat{F}\left(c\right)}\geq \frac{f(c)}{F(c)}$.

Definition 3 implies that, conditional on any maximum level of the expected cost, a 3PL is more likely to observe a lower expected cost from *F*(*c*) than from $\hat{F}\left(c\right)$. Next, we introduce the definition of the regular condition below [42].

Definition 4. *For* $c\in [\underline{c},\bar{c}]$, *if* $\frac{F(c)}{f(c)}$ *is increasing in c*, *then F*(*c*) *satisfies the regularity condition*.

Definition 4 implies that *F*(*c*) is a log-concave function. For example, the frequently used distributions like uniform, normal and exponential distributions can satisfy the regularity condition.

Comparing Propositions 2 and 3, we have the following proposition.

Proposition 4. *Given the optimal parameters* (Δ^*E*^, *β*^*E*^) *and* (Δ^*O*^, *β*^*O*^) *of ESA*-*CC and OSA*-*CC*, *respectively*, *if F*_*h*_(*c*) *satisfies the regularity condition and F*_*l*_(*c*) *is a distributional upgrade of F*_*h*_(*c*), *then we have* Δ^*O*^ < Δ^*E*^, *and* *β*^*O*^ > *β*^*E*^.

Proposition 4 implies that to maximize the ex ante expected profit, the 4PL should reduce the cost advantage value of a 3PL with low delivery risk in OSA-CC than that in ESA-CC, i.e., Δ^O^ < Δ^E^. Intuitively, a smaller Δ^O^ makes a 3PL with high delivery risk have a higher chance to win the reverse auction, and thus under-rewards 3PLs with low delivery risk by reducing the information rent induced by the high delivery risk 3PLs. In addition, the optimal cost-sharing parameter shall be higher in OSA-CC than that in ESA-CC, i.e., *β*^O^ > *β*^E^. Intuitively, setting a higher *β*^O^, the 4PL would derive a greater fraction of the extra amount of expected profit from the cost-reduction effort of a 3PL. Yet a larger *β*^O^ would also make the 3PL exert less effort to reduce the future cost, which then reduces the extra amount of expected profit created by the 3PL. Hence, the 4PL could maximize her ex ante expected profit by manipulating *β* and Δ.

## Numerical experiments

In this section, numerical examples are presented to illustrate the research problem we studied and to provide useful insights for the 4PL. We assume that the standard deviation of the unpredictable cost is *σ* = 1. The cumulative distribution functions of the expected cost for 3PLs of high and low delivery risks are assumed to be *F*_*h*_(*c*) = *F*_*l*_(*c*) = *c*, *c* ∈ [0, 1]. The 4PL derives *v* = 3 if the delivery succeeds, and loses *z* = 3 if the delivery risk strikes. The scoring function is $S\left(b,t\right)=b-w\sqrt{t}$, *w* > 0, and the disutility function is $h\left(e\right)=\frac{1}{2}e^{2}$.

### Sensitive analysis of ESA-CC

When *β* = 0, the payment scheme becomes *p* = *b*. In this case, ESA-CC degenerates to *efficient scoring auction* (ESA), which is adopted by Chen et al. [24]. When Δ = 0, ESA-CC degenerates to standard *efficient reverse auction with cost-sharing contract* (ERA-CC). When *β* = 0 and Δ = 0, ESA-CC degenerates to standard *efficient reverse auction* (ERA).

**Fig 2:**
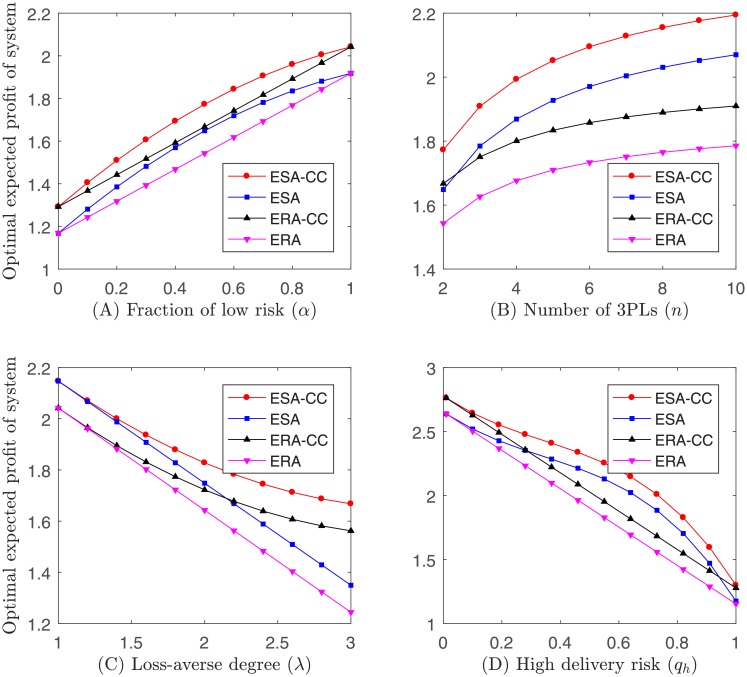
Impact of parameters on optimal ex ante expected profit of the system.

Example 1. Let *q*_*l*_ = 1/4, *q*_*h*_ = 1/2, λ = 2.25 and *n* = 2, given a fixed set of *α* ∈ {0, 0.1, 0.2, 0.3, 0.4, 0.5, 0.6, 0.7, 0.8, 0.9, 1}, the optimal ex ante expected profit of the system is shown in Fig 2A. Let *q*_*l*_ = 1/4, *q*_*h*_ = 1/2, λ = 2.25 and *α* = 1/2, given a fixed set of *n* ∈ {2, 3, 4, 5, 6, 7, 8, 9, 10}, the optimal ex ante expected profit of the system is shown in Fig 2B. Let *q*_*l*_ = 1/4, *q*_*h*_ = 1/2, *n* = 2 and *α* = 1/2, given a fixed set of λ ∈ {1, 1.2, 1.4, 1.6, 1.8, 2, 2.2, 2.4, 2.6, 2.8, 3}, the optimal ex ante expected profit of the system is shown in Fig 2C. Let *q*_*l*_ = 0.01, λ = 2.25, *n* = 2 and *α* = 1/2, given a fixed set of *q*_*h*_ ∈ {0.01, 0.1, 0.19, 0.28, 0.37, 0.46, 0.55, 0.64, 0.73, 0.82, 0.91, 1}, the optimal ex ante expected profit of the system is shown in Fig 2D.

Observation 1. *The fraction of low delivery risk and the number of 3PLs have a positive impact on the optimal ex ante expected profit of the system*. *Yet*, *the loss-averse attitude and the high delivery risk have a negative impact on the optimal ex ante expected profit of the system*.

Intuitively, (A) when *α* increases, 3PLs are more likely to have a low delivery risk, which would benefit the 4PL and system. (B) When *n* increases, other things being equal, the number of 3PLs with low delivery risk increases, and thereby the expected profit of the 4PL and system shall increase. (C) When λ increases, the 3PL’s expected utility decreases, and thus the expected profit of the system decreases. The analysis indicates that the loss-averse behavior of 3PLs would have adverse effect on the optimal ex ante expected profit of the system. (D) When *q*_*h*_ increases, 3PLs with low delivery risk shall be over-rewarded to promote competitions, which would reduce the expected profit of the system.

Observation 2. *ESA*-*CC* *performs better than ESA*, *ERA*-*CC* *and ERA*.

Noting that ESA-CC is a general case of ESA, ERA-CC and ERA, and thereby shall be the best choice for the 4PL to maximize the expected profit of the system.

### Sensitive analysis of OSA-CC

**Fig 3:**
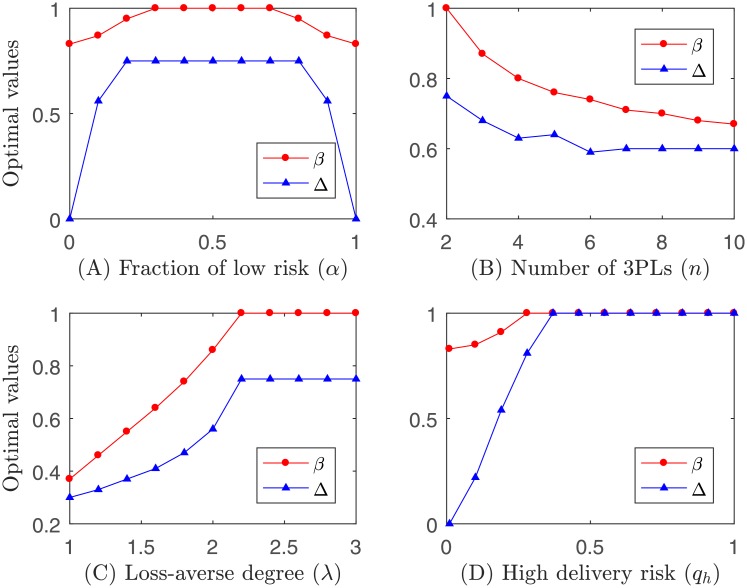
Impact of parameters on optimal *β* and Δ.

Example 2. Let *q*_*l*_ = 1/4, *q*_*h*_ = 1/2, λ = 2.25 and *n* = 2, given a fixed set of *α* ∈ {0, 0.1, 0.2, 0.3, 0.4, 0.5, 0.6, 0.7, 0.8, 0.9, 1}, the optimal *β* and Δ are shown in Fig 3A. Let *q*_*l*_ = 1/4, *q*_*h*_ = 1/2, λ = 2.25 and *α* = 1/2, given a fixed set of *n* ∈ {2, 3, 4, 5, 6, 7, 8, 9, 10}, the optimal *β* and Δ are shown in Fig 3B. Let *q*_*l*_ = 1/4, *q*_*h*_ = 1/2, *n* = 2 and *α* = 1/2, given a fixed set of λ ∈ {1, 1.2, 1.4, 1.6, 1.8, 2, 2.2, 2.4, 2.6, 2.8, 3}, the optimal *β* and Δ are shown in Fig 3C. Let *q*_*l*_ = 0.01, λ = 2.25, *n* = 2 and *α* = 1/2, given a fixed set of *q*_*h*_ ∈ {0.01, 0.1, 0.19, 0.28, 0.37, 0.46, 0.55, 0.64, 0.73, 0.82, 0.91, 1}, the optimal *β* and Δ are shown in Fig 3D.

Observation 3. *The optimal β and* Δ *first increase and then decrease in the fraction of low delivery risk*. *The optimal β and* Δ *decrease as the number of 3PLs increases*, *but increase as the loss-averse attitude of 3PLs is intensified or as the high delivery risk of 3PLs increases*.

Intuitively, (A) when *α* = 0 or *α* = 1, the 4PL faces a traditional auction and Δ = 0. In this case, *β* shall be the lowest. When 0 < *α* < 1, the 4PL has to manipulate Δ and *β* to achieve the highest expected profit. (B) When *n* increases, the number of 3PLs with low delivery risk increases, and thus there is no need to induce competition of 3PLs with low delivery risk. Hence, we observe that Δ decreases. In this case, the 4PL could induce 3PLs to exert efforts by decreasing *β*. (C) When λ increases, the increased loss aversion of 3PLs shall be neutralized by sharing less risk of cost uncertainty, and thus *β* would increase until its bounded value. In this case, Δ shall be increased to promote competitions across 3PLs. (D) When *q*_*h*_ increases, the 4PL has to reward 3PLs with low delivery risk to increase the competition, and could derive a higher fraction of the extra amount of expected profit from the cost-reduction effort of the winning 3PL. Hence, Δ and *β* would increase until the bounded value.

Observation 4. *The trend of optimal β and* Δ *follows the same pattern*.

The interpretation of Observation 4 follows the same logic as in Proposition 4.

When *β* = 0, OSA-CC degenerates to *optimal scoring auction* (OSA), which is adopted by Chen et al. [24]. When Δ = 0, OSA-CC degenerates to standard *optimal reverse auction with cost-sharing contract* (ORA-CC). When *β* = 0 and Δ = 0, OSA-CC degenerates to standard *optimal reverse auction* (ORA).

**Fig 4:**
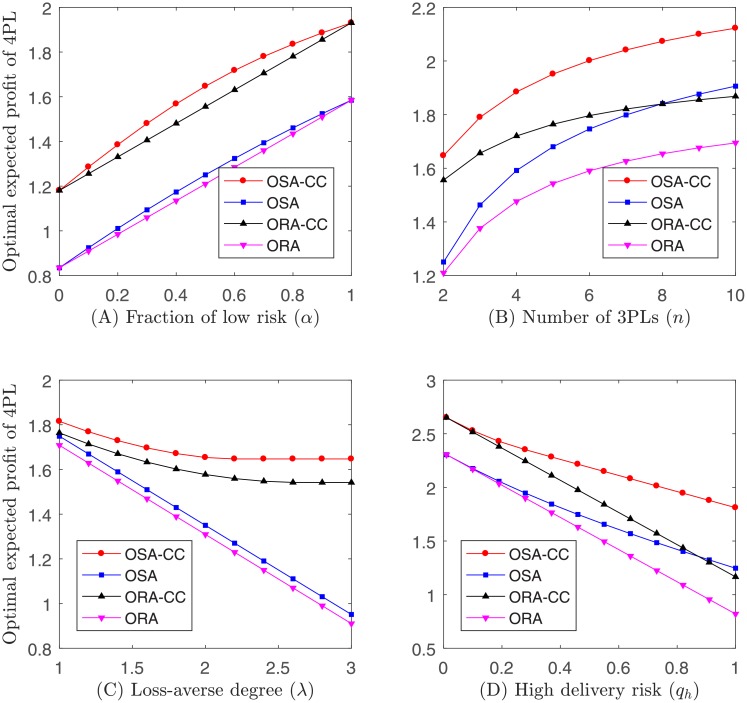
Impact of parameters on optimal ex ante expected profit of 4PL.

Example 3. Let *q*_*l*_ = 1/4, *q*_*h*_ = 1/2, λ = 2.25 and *n* = 2, given a fixed set of *α* ∈ {0, 0.1, 0.2, 0.3, 0.4, 0.5, 0.6, 0.7, 0.8, 0.9, 1}, the optimal ex ante expected profit of 4PL is shown in Fig 4A. Let *q*_*l*_ = 1/4, *q*_*h*_ = 1/2, λ = 2.25 and *α* = 1/2, given a fixed set of *n* ∈ {2, 3, 4, 5, 6, 7, 8, 9, 10}, the optimal ex ante expected profit of 4PL is shown in Fig 4B. Let *q*_*l*_ = 1/4, *q*_*h*_ = 1/2, *n* = 2 and *α* = 1/2, given a fixed set of λ ∈ {1, 1.2, 1.4, 1.6, 1.8, 2, 2.2, 2.4, 2.6, 2.8, 3}, the optimal ex ante expected profit of 4PL is shown in Fig 4C. Let *q*_*l*_ = 0.01, λ = 2.25, *n* = 2 and *α* = 1/2, given a fixed set of *q*_*h*_ ∈ {0.01, 0.1, 0.19, 0.28, 0.37, 0.46, 0.55, 0.64, 0.73, 0.82, 0.91, 1}, the optimal ex ante expected profit of 4PL is shown in Fig 4D.

Observation 5. *The impact of parameters on the optimal ex ante expected profit of 4PL is similar to that on the optimal ex ante expected profit of the system*.

The interpretation of Observation 5 follows the same logic as in Observation 1.

Observation 6. *OSA*-*CC performs better than OSA*, *ORA*-*CC* *and ORA*.

The interpretation of Observation 6 follows the same logic as in Observation 2.

## Conclusions

4PL could assemble the resources, capabilities, and technology of its own organization and other organizations to design, build and run comprehensive supply chain solutions. Adopting multi-attribute reverse auctions to select a 3PL forming a longer-term strategic partnership could improve the efficiency of the transportation services for the 4PL. Because of uncertainties that surround the process of logistics, such as the fluctuating cost of energy or labor, 3PLs generally face cost uncertainty and would involve loss averse behaviors during the decision process. Also, delivery failures like traffic jam and technology malfunctions may occur unintentionally for reasons beyond the 3PLs’ control. Considering such incomplete attributes, i.e., the cost uncertainty and delivery risk, this study addresses a real management problem faced by a 4PL who designs an auction mechanism to purchase transportation services from multiple loss-averse 3PLs for satisfying the market demand of clients.

Based on the prospect theory and game theory, the bid decision of 3PLs is investigated and analyzed. We find that 3PLs would bid higher as the loss-averse degree becomes intensified. Integrating the contingent penalty and cost-sharing contract into multi-attribute reverse auctions, we propose the novel efficient and optimal scoring auctions with cost-sharing contract depending on whether the 4PL is willing to cooperate or not. Theoretical analysis and numerical results show the distinct advantage of the proposed mechanisms. They could be a useful tool for the 4PL to manage the cost uncertainty and delivery risk of 3PLs. Sensitive analysis shows that the loss-averse behavior of 3PLs would have adverse effect on the optimal ex ante expected profit of the system and the 4PL. Our work provides an effective solution method for real applications of the 4PL to select a strategic 3PL through reverse auctions, which could improve the operational efficiency of the 4PL and would further benefit the logistics industry.

By relaxing some of the assumptions, several issues may be interesting for future research. First, the loss-averse parameter of 3PLs could be assumed to follow some publicly known distribution. In this case, our model could provide an approximation for 3PLs to determine their bids if the fixed loss-averse parameter is replaced by the mean of the loss-averse parameter. Second, the loss-averse parameter could be assumed to be known only to the individual 3PL (private information). In this situation, higher dimensional asymmetric information would be involved in the analysis of the mechanism design problem. Hence it becomes difficult to derive closed-form solutions of 3PLs’ bid decisions and the 4PL’s mechanism problem.

## Appendix 1. Proofs

### Proof of Lemma 1

*Proof*. Tanking the first and second order derivative of Eq (2) with respect to *e*, we obtain *dπ*_3*PL*_(*e*; *b*, *t*, *c*, *q*, *β*)/*de* = 1 − *β* − 2*ae* and *d*^2^ *π*_3*PL*_(*e*; *b*, *t*, *c*, *q*, *β*)/*de*^2^ = −2*a* < 0. Hence we shall see that $e^{*}=\frac{1-\beta }{2a}$ maximizes Eq (2), and e* is decreasing in *β*.

### Proof of Lemma 2

*Proof*. Taking the first and second order derivative of *S*(*b*, *t*) with respect to *t*, we have *dS*(*b*, *t*)/*dt* = *q* − Λ′(*t*) and *d*^2^ *S*(*b*, *t*)/*dt*^2^ = −Λ″(*t*)>0. Hence we see that t*(*q*) = Λ′^−1^(*q*) minimizes *S*(*b*, *t*). In addition, since *q* = Λ′(*t*), the left-hand side of the equation is increasing in *q* and is independent of *t*, but the right-hand side of the equation is decreasing in *t*, hence we shall see that t*(*q*) is decreasing in *q*.

### Proof of Proposition 1

*Proof*. Based on Eq (5a), by envelope theorem, we have

$$\frac{dU_{H}\left(b;c,q_{h},t^{*}\left(\cdot \right),e^{*}\left(\cdot \right),\beta \right)}{dc}=\frac{\partial U_{H}}{\partial c}=-\left(1-\beta \right)\theta_{h}\left(c\right).$$ (10)

Note that the ex ante expected profit of a high delivery risk 3PL with the highest expected cost is zero, i.e., $U_{H}\left(b;\bar{c},q_{h},t^{*}\left(\cdot \right),e^{*}\left(\cdot \right),\beta \right)=0$. Performing the integral operation on *c* for each side of Eq (10), we see

$$U_{H}\left(b;c,q_{h},t^{*}\left(\cdot \right),e^{*}\left(\cdot \right),\beta \right)=\left(1-\beta \right)\int_{c}^{\bar{c}}\theta_{h}\left(x\right)dx$$ (11)

From Eqs (5a) and (11), we have $[\left(1-\beta \right)\left(b-c-t^{*}\left(q_{h}\right)q_{h}-\left(\lambda -1\right)\sigma /\sqrt{2\pi }\right)+k^{*}\left(\beta \right)]\theta_{h}\left(c\right)=\left(1-\beta \right)\int_{c}^{\bar{c}}\theta_{h}\left(x\right)dx$. Hence, we derive Eq (6a).

Following the same logic, according to Eq (5b), we can obtain Eq (6b).

For sufficiency, we shall show that *b*_*h*_ and *b*_*l*_ are global maximizers of Eqs (5a) and (5b). In other words, given other 3PLs follow *b*_*h*_ or *b*_*l*_, any 3PL will not deviate from the presumed bidding strategy. Suppose that a 3PL with expected cost *c* would submit a different bid price ${\hat{b}}_{h}$. For any $\hat{c}\in \left[\underline{c},\bar{c}\right]$, let ${\hat{b}}_{h}=b_{h}\left(\hat{c};q_{l},t^{*}\left(\cdot \right),e^{*}\left(\cdot \right),\beta \right)$. The difference of his ex ante expected profit between the truthful and untruthful bids is $U_{H}\left(b_{h}\left(\hat{c};q_{l},t^{*}\left(\cdot \right),e^{*}\left(\cdot \right),\beta \right);c,q_{h},t^{*}\left(\cdot \right),e^{*}\left(\cdot \right),\beta \right)-U_{H}\left(b_{h}\left(c;q_{l},t^{*}\left(\cdot \right),e^{*}\left(\cdot \right),\beta \right);c,q_{h},t^{*}\left(\cdot \right),e^{*}\left(\cdot \right),\beta \right)=\left(\hat{c}-c\right)\theta_{h}\left(\hat{c}\right)-\int_{c}^{\hat{c}}\theta_{h}\left(x\right)dx=\left(c-\hat{c}\right)\left(\theta_{h}\left(\hat{c}\right)-\theta_{h}\left(\xi \right)\right)$, where $c<\xi <\hat{c}$, and the last equality is based on the mean value theorem of integral. Noting that *θ*_*h*_(⋅) is a decreasing function, the difference of the ex ante expected profit is negative for any $\hat{c}\neq c$. Hence, bidding *b*_*h*_ could globally maximize Eq (5a). Similar arguments apply to Eq (5b).

### Proof of Corollary 1

*Proof*. Corollary 1 is a direct consequence of Proposition 1.

### Proof of Corollary 2

*Proof*. Corollary 2 is a direct consequence of Proposition 1.

### Proof of Proposition 2

*Proof*. Noting that *θ*_*l*_(*c*) = *θ*_*h*_(*c* − Δ), we have $\frac{d\theta_{h}\left(c\right)}{d\Delta }\;|{\;}_{c=c-\Delta }=-\frac{\alpha }{1-\alpha }\frac{f_{l}\left(c\right)}{f_{h}\left(c-\Delta \right)}\frac{d\theta_{l}\left(c\right)}{d\Delta }$, where $\frac{d\theta_{l}\left(c\right)}{d\Delta }=0$ when $c<\underline{c}+\Delta$ and $\frac{d\theta_{h}\left(c\right)}{d\Delta }=0$ when $c>\bar{c}-\Delta$. Taking the first-order partial derivative of Γ(Δ, *β*) with respect to Δ, we derive

$$\begin{array}{ccc} \frac{\partial \varphi (\Delta ,\beta )}{\partial \Delta } & = & -n\alpha \int_{\underline{c}}^{\bar{c}}\left(c+q_{l}z\right)\frac{d\theta_{l}\left(c\right)}{d\Delta }f_{l}\left(c\right)dc-n\left(1-\alpha \right)\int_{\underline{c}}^{\bar{c}}\left(c+q_{h}z\right)\frac{d\theta_{h}\left(c\right)}{d\Delta }f_{h}\left(c\right)dc\\ & = & -n\alpha \int_{\underline{c}+\Delta }^{\bar{c}}\left(c+q_{l}z\right)\frac{d\theta_{l}\left(c\right)}{d\Delta }f_{l}\left(c\right)dc-n\left(1-\alpha \right)\int_{\underline{c}}^{\bar{c}-\Delta }\left(c+q_{h}z\right)\frac{d\theta_{h}\left(c\right)}{d\Delta }f_{h}\left(c\right)dc,\\ & = & n\alpha \int_{\underline{c}+\Delta }^{\bar{c}}\left(q_{h}z-q_{l}z-\Delta \right)\frac{d\theta_{l}\left(c\right)}{d\Delta }f_{l}\left(c\right)dc. \end{array}$$

where the last equality is adopting the integration by substitution. Obviously, ∂*φ*(Δ, *β*)/∂Δ > 0 if Δ < (*q*_*h*_ − *q*_*l*_)*z*, ∂*φ*(Δ, *β*)/∂Δ < 0 if Δ > (*q*_*h*_ − *q*_*l*_)*z* and ∂*φ*(Δ, *β*)/∂Δ = 0 if Δ = (*q*_*h*_ − *q*_*l*_)*z*. Hence, we have ∂^2^ *φ*(Δ, *β*)/∂Δ^2^ < 0 and ∂^2^ *φ*(Δ, *β*)/(∂Δ∂*β*) = 0.

In addition, we have $\frac{\partial \varphi (\Delta ,\beta )}{\partial \beta }=-\frac{\beta }{2a}+\left(\lambda -1\right)\sigma /\sqrt{2\pi }$ and $\frac{\partial^{2}\varphi \left(\Delta ,\beta \right)}{\partial \beta^{2}}=-\frac{1}{2a}<0$. By checking the optimality condition, we see that Δ = (*q*_*h*_ − *q*_*l*_)*z* and $\beta =2a\left(\lambda -1\right)\sigma /\sqrt{2\pi }$ can globally maximize Γ(Δ, *β*).

### Proof of Corollary 3

*Proof*. Corollary 3 is a direct consequence of Proposition 2.

### Proof of Proposition 3

*Proof*. Taking the first-order partial derivative of Eq (8) with respect to *β*, we have

$$\frac{\partial ϒ(\Delta ,\beta )}{\partial \beta }=-\frac{\beta }{2a}+\left(\lambda -1\right)\sigma /\sqrt{2\pi }+n\alpha \int_{\underline{c}}^{\bar{c}}F_{l}\left(c\right)\theta_{l}\left(c\right)dc+n\alpha \int_{\bar{c}-\Delta }^{\bar{c}}\theta_{h}\left(c\right)dc+n\left(1-\alpha \right)\int_{\underline{c}}^{\bar{c}}F_{h}\left(c\right)\theta_{h}\left(c\right)dc.$$

Noting that $\frac{d\theta_{l}\left(c\right)}{d\Delta }=0$ when $c<\underline{c}+\Delta$, $\frac{d\theta_{h}\left(c\right)}{d\Delta }=0$ when $c>\bar{c}-\Delta$, and $\frac{d\theta_{h}\left(c\right)}{d\Delta }\;|{\;}_{c=c-\Delta }=-\frac{\alpha }{1-\alpha }\frac{f_{l}\left(c\right)}{f_{h}\left(c-\Delta \right)}\frac{d\theta_{l}\left(c\right)}{d\Delta }$, the first-order partial derivative of Eq (8) with respect to Δ can be derived as

$$\begin{array}{ccc} \frac{\partial ϒ(\Delta ,\beta )}{\partial \Delta } & = & -n\alpha \int_{\underline{c}+\Delta }^{\bar{c}}[q_{l}z+c+\left(1-\beta \right)\frac{F_{l}\left(c\right)}{f_{l}\left(c\right)}]\frac{d\theta_{l}\left(c\right)}{d\Delta }f_{l}\left(c\right)dc-n\alpha \left(1-\beta \right)\theta_{h}\left(\bar{c}-\Delta \right)\\ & & -n\left(1-\alpha \right)\int_{\underline{c}}^{\bar{c}-\Delta }[q_{h}z+c+\left(1-\beta \right)\frac{F_{h}\left(c\right)}{f_{h}\left(c\right)}]\frac{d\theta_{h}\left(c\right)}{d\Delta }f_{h}\left(c\right)dc\\ & = & -n\alpha \int_{\underline{c}+\Delta }^{\bar{c}}[q_{l}z+c+\left(1-\beta \right)\frac{F_{l}\left(c\right)}{f_{l}\left(c\right)}]\frac{d\theta_{l}\left(c\right)}{d\Delta }f_{l}\left(c\right)dc-n\alpha \left(1-\beta \right)\theta_{h}\left(\bar{c}-\Delta \right)\\ & & -n\left(1-\alpha \right)\int_{\underline{c}+\Delta }^{\bar{c}}[q_{h}z+c-\Delta +\left(1-\beta \right)\frac{F_{h}\left(c-\Delta \right)}{f_{h}\left(c-\Delta \right)}]\frac{d\theta_{h}\left(c-\Delta \right)}{d\Delta }f_{h}\left(c-\Delta \right)dc\\ & = & n\alpha \int_{\underline{c}+\Delta }^{\bar{c}}[\left(q_{h}-q_{l}\right)z-\Delta +\left(1-\beta \right)\left[\frac{F_{h}\left(c-\Delta \right)}{f_{h}\left(c-\Delta \right)}-\frac{F_{l}\left(c\right)}{f_{l}\left(c\right)}\right]]\frac{d\theta_{l}\left(c\right)}{d\Delta }f_{l}\left(c\right)dc\\ & & -n\alpha \left(1-\beta \right)\theta_{h}\left(\bar{c}-\Delta \right) \end{array}$$

Obviously, Eq (8) can be maximized at $\frac{\partial ϒ(\Delta ,\beta )}{\partial \beta }=0$ and $\frac{\partial ϒ(\Delta ,\beta )}{\partial \Delta }=0$, or at the corner points. Noting that $\frac{\partial ϒ(\Delta ,\beta )}{\partial \beta }{|}_{\beta =0}>0$ and $\frac{\partial ϒ(\Delta ,\beta )}{\partial \Delta }{|}_{\Delta =\bar{c}-\underline{c}}<0$, we see that *β* = 0 and $\Delta =\bar{c}-\underline{c}$ cannot form a corner solution. Therefore, the corner solution might be (0, 1), i.e., Δ = 0 and *β* = 1.

### Proof of Proposition 4

*Proof*. For $c\in [\underline{c}+\Delta ,\bar{c}]$, given that *F*_*h*_(*c*) satisfies the regularity condition and *F*_*l*_(*c*) is a distributional upgrade of *F*_*h*_(*c*), we have

$$\frac{F_{h}\left(c-\Delta \right)}{f_{h}\left(c-\Delta \right)}<\frac{F_{h}\left(c\right)}{f_{h}\left(c\right)}\leq \frac{F_{l}\left(c\right)}{f_{l}\left(c\right)}.$$

Since $\frac{d\theta_{l}\left(c\right)}{dc}>0$ and $\theta_{h}\left(\bar{c}-\Delta \right)>0$, we see that ∂*Υ*(Δ, *β*)/∂Δ < 0 for all Δ ≥ *z*(*q*_*h*_ − *q*_*l*_). Therefore, we shall see that Δ^*O*^ < Δ^*E*^. Similarly, we could see that *β*^*O*^ > *β*^*E*^.

## Appendix 2. The Derivation of the 4PL’s ex ante expected profit

The 4PL’s ex ante expected profit from an individual 3PL is equal to the ex ante expected profit of the system for choosing the 3PL minus the 3PL’s ex ante expected profit, i.e.,

$$\left[v-c-q_{j}z+e^{*}\left(\beta \right)-h\left(e^{*}\left(\beta \right)\right)-\left(1-\beta \right)\left(\lambda -1\right)\sigma /\sqrt{2\pi }\right]\theta_{j}\left(c\right)-U\left(b_{j},c,q_{j},t^{*}\left(\cdot \right),e^{*}\left(\cdot \right),\beta \right),$$

where *j* ∈ {*h*, *l*}. Applying Eq (11), we have

$$\begin{array}{ccc} & & \alpha E[\left[v-c-q_{l}z+e^{*}\left(\beta \right)-h\left(e^{*}\left(\beta \right)\right)-\frac{(1-\beta )(\lambda -1)\sigma }{\sqrt{2\pi }}\right]\theta_{l}\left(c\right)-U_{L}\left(b_{l},c,q_{l},t^{*}\left(\cdot \right),e^{*}\left(\cdot \right),\beta \right)]\\ & & +(1-\alpha )\cdot \\ & & E[\left[v-c-q_{h}z+e^{*}\left(\beta \right)-h\left(e^{*}\left(\beta \right)\right)-\frac{(1-\beta )(\lambda -1)\sigma }{\sqrt{2\pi }}\right]\theta_{h}\left(c\right)-U_{H}\left(b_{h},c,q_{h},t^{*}\left(\cdot \right),e^{*}\left(\cdot \right),\beta \right)]\\ & = & \alpha \int_{\underline{c}}^{\bar{c}}[\left(v-c-q_{l}z+e^{*}\left(\beta \right)-h\left(e^{*}\left(\beta \right)\right)\right)\theta_{l}\left(c\right)-\left(1-\beta \right)(\int_{c}^{\bar{c}}\theta_{l}\left(x\right)dx-\int_{c^{*}}^{\bar{c}}\theta_{h}\left(x\right)dx)]f_{l}\left(c\right)dc\\ & & +\left(1-\alpha \right)\int_{\underline{c}}^{\bar{c}}[\left(v-c-q_{h}z+e^{*}\left(\beta \right)-h\left(e^{*}\left(\beta \right)\right)\right)\theta_{h}\left(c\right)-\left(1-\beta \right)\int_{c}^{\bar{c}}\theta_{h}\left(x\right)dx]f_{h}\left(c\right)dc\\ & = & \frac{1}{n}\left[v+e^{*}\left(\beta \right)-h\left(e^{*}\left(\beta \right)\right)-\frac{(1-\beta )(\lambda -1)\sigma }{\sqrt{2\pi }}\right]-\alpha \left(1-\beta \right)\int_{\bar{c}-\Delta }^{\bar{c}}\theta_{h}\left(c\right)dc\\ & & -\alpha \int_{\underline{c}}^{\bar{c}}[q_{l}z+c+\left(1-\beta \right)\frac{F_{l}\left(c\right)}{f_{l}\left(c\right)}]\theta_{l}\left(c\right)dc-\left(1-\alpha \right)\int_{\underline{c}}^{\bar{c}}[q_{h}z+c+\left(1-\beta \right)\frac{F_{h}\left(c\right)}{f_{h}\left(c\right)}]\theta_{h}\left(c\right)dc \end{array}$$ (12)

where the first equality is due to $\alpha \int_{\underline{c}}^{\bar{c}}\theta_{l}\left(c\right)f_{l}\left(c\right)dc+\left(1-\alpha \right)\int_{\underline{c}}^{\bar{c}}\theta_{h}\left(c\right)f_{h}\left(c\right)dc=1/n$, and the second equality is achieved by exchanging the integration order. Multiplying Eq (12) by *n*, we derive the 4PL’s ex ante expected profit.
